# EPG combined with micro-CT and video recording reveals new insights on the feeding behavior of *Philaenus spumarius*

**DOI:** 10.1371/journal.pone.0199154

**Published:** 2018-07-17

**Authors:** Daniele Cornara, Elisa Garzo, Marina Morente, Aranzazu Moreno, Javier Alba-Tercedor, Alberto Fereres

**Affiliations:** 1 Instituto de Ciencias Agrarias, Consejo Superior de Investigaciones Cientificas, ICA-CSIC, Madrid, Spain; 2 Department of Zoology, Faculty of Sciences, University of Granada, Campus de Fuentenueva, Granada, Spain; University of Idaho, UNITED STATES

## Abstract

The meadow spittlebug *Philaenus spumarius* plays a key role in the transmission of the bacterium *Xylella fastidiosa* to olive in Apulia (South Italy). Currently, available data on *P*. *spumarius* feeding behavior is limited, and a real-time observation of the different steps involved in stylet insertion, exploratory probes, and ingestion, has never been carried out. Therefore, we performed an EPG-assisted characterization of *P*. *spumarius* female feeding behavior on olive, in order to detect and analyze the main EPG waveforms describing their amplitude, frequency, voltage level, and electrical origin of the traces during stylet penetration in plant tissues. Thereafter, each of the main waveforms was correlated with specific biological activities, through video recording and analysis of excretion by adults and excretion/secretion by nymphs. Furthermore, the specific stylet tips position within the plant tissues during each of the waveforms observed was assessed by microcomputer tomography (micro-CT). Additional EPG-recordings were carried out with males of *P*. *spumarius* on olive, in order to assess possible sex-related differences. *P*. *spumarius* feeding behavior can be described by five main distinct waveforms: C (pathway), Xc (xylem contact/pre-ingestion), Xi (xylem sap ingestion), R (resting), N (interruption within xylem phase). Compared to males, females require shorter time to begin the first probe, and their Xi phase is significantly longer. Furthermore, considering the single waveform events, males on olive exhibit longer np and R compared to females.

## Introduction

One of the primary concerns of the global fruit industry is a group of systemic plant pathogens, i.e. virus, viroids, phytoplasmas and bacteria, for which there are not remedies once the plant is infected. The spread of these pathogens after the introduction into a new location generally relies on native or introduced vectors [1, 2, 3, 4]. Vector abundance, activity, and behavior, are key factors for the transmission of insect-borne plant pathogens [5]. Furthermore, considering pathosystems where the pathogen is transmitted without strain specificity among vectors, as in the case of the plant-pathogenic bacterium *Xylella fastidiosa* Wells (1987) [6], a detailed knowledge of vector feeding behavior is of paramount importance. Only xylem-sap feeding insects are able to transmit *X*. *fastidiosa* [7]. Xylem sap feeding is a characteristic shared by three *taxa* belonging to the order Hemiptera: Cicadellidae Cicadellinae (aka sharpshooters), Cercopoidea (froghoppers or spittlebugs), and Cicadoidea (cicadas) [8]. Although occasional xylem sap feeding by insects that generally prefer other tissues may occur [9, 10, 11], the “non-xylem specialists” are unable to transmit the bacterium [12]. To date, in Europe, the meadow spittlebug *Philaenus spumarius* L. (1758) (Hemiptera: Aphrophoridae) has been proven to be the main vector of *X*. *fastidiosa* to olive and likely other host plants [13, 14]. *P*. *spumarius* is a xylem feeder, either as nymph or adult. The spittlebug ingests considerable amount of sap from the main transpiration stream without causing vessel cavitation, overcoming dramatically high tension reaching -10 bars or more, and showing a mean excretion rate of 280 times its body weight in 24 hours [15, 16, 17, 18, 19, 20]. The association with symbionts potentially relaxes the severe dietary limitations related to xylem sap feeding, due to the poor nutritional value of the xylem sap and the cost associated with the process [21, 22].

All the published data currently available on *P*. *spumarius* feeding behavior have been produced with methods that only furnish snapshots and indirect evidence of the entire process, because during a probe the stylets are inside the plant tissue, and insect activity can only be inferred. To the best of our knowledge, a real-time characterization of *P*. *spumarius* feeding behavior, only obtainable with the Electrical Penetration Graph (EPG) technique is missing. Electrical Penetration Graph is a technology devised by McLean and Kinsey [23] based on alternating current (thus AC-EPG), then modified by Tjallingii [24] using direct current (thus DC-EPG), and then blended by Backus and Bennett [25] using selectable AC or DC features and a variable input resistance. EPG is considered an essential tool in research on feeding behavior and pathogens transmission by piercing-sucking insects [26]. In EPG, biopotentials and electrical resistances transform the constantly applied electrical input in a variable voltage output, graphed in waveforms [27]. Eventually, each of these waveforms represents a precise activity of the insect feeding behavior. While this tool has been used for the characterization of the feeding behavior of over 50 species of hemipterans by the 1990’s [28], since then, only two (primarily quantitative) EPG studies on Aphrophoridae (spittlebugs) have been published. Such studies were mainly aimed at determining EPG parameters viable for host-range assessment more than at characterizing spittlebugs feeding behavior, and rely on the similarities of the observed waveforms with those previously described for other xylem feeders [29, 30]. Moreover, a detailed knowledge of *P*. *spumarius* feeding behavior could furnish useful data in order to understand the intimate relationship occurring between the meadow spittlebug and *X*. *fastidiosa*.

The main goals of this study were: (1) to characterize the EPG waveforms produced by *P*. *spumarius* on olive; (2) to understand and define the biological meaning of the waveforms, by correlating EPG signals with histological analysis of tissues penetrated by the spittlebugs and through video-recording of the excretion process in adults and excretion/secretion in nymphs; and (3) to identify sex-related differences in the feeding behavior of *P*. *spumarius*.

## Materials and methods

### Collection and rearing of *P*. *spumarius* nymphs and adults

*P*. *spumarius* nymphs were collected in Osuna (Sevilla, Spain) on *Bidens* sp. L. (1753), *Sonchus* sp. L. (1753), *Taraxacum* sp. (Wigg, 1780), *Cirsium* sp. (Miller, 1754), *Borago officinalis* L., *Calendula* sp. L. (1753), and *Scolymus hispanicus* L., and reared on one month old *Sonchus oleraceus* L. (1753) plants until adulthood. Both nymphs and adults were reared in the controlled-environmental facilities of Instituto de Ciencias Agrarias-Consejo Superior de Investigaciones Cientificas (ICA-CSIC, Madrid, Spain) in a walk-in growth chamber at 18°C night and 23°C day temperature, humidity of ca. 60%, and photoperiod 14:10 L:D. The adults were transferred in groups of ten per plant to two weeks old vetch (*Vicia sativa* L.) plants, with the host plant replaced every ten days. No pre-screening tests to assess the infectivity of *P*. *spumarius* to *X*. *fastidiosa* have been performed. Nevertheless, since i) nymphs were collected from *X*. *fastidiosa*-free areas; ii) bacterial cells are shed with each molt [31]; the probability that the adults used in this study were infective is negligible.

### Plant rearing

The plants used for the EPG were three months olive cuttings cv. Picual, rooted in vermiculite in a humid chamber, and transplanted in 3L pots with universal soil, sand and vermiculite (6:3:2). Plants were grown in a greenhouse (ICA-CSIC Madrid) (25°C during day, 18°C during night, no humidity and photoperiod control) and water-fertilized weekly with a nutritional complex 20-20-20 (N:P:K) of Nutrichem 60 fertilizer (Miller Chemical & Fertilizer. Hanover, PA, USA) (1 g/l). *S*. *oleraceus* and vetch plants used for *P*. *spumarius* rearing were seedlings germinated and maintained in a growth chamber (25/18 °C day/night temperature, 60% humidity, 16/8 L/D photoperiod) in 5L pots filled with universal soil, and water-fertilized every two days with the nutritional complex described above.

### Characterization of the EPG waveforms of *P*. *spumarius* adults

The feeding behavior of *P*. *spumarius* on olive (*Olea europaea*, L. 1753) plants was characterized by EPG, using 20 spittlebug adult females, within ten days after adult emergence. This first experiment allowed us to identify EPG signals related to the main feeding phases, and describe the main characteristics of the waveforms obtained (relative amplitude; frequency; voltage level, extra- or intracellular; main electrical origin, emf (electromotive force) or R (resistance)). Adult females were moved to olive plants two days before the EPG recording for acclimation, and kept in the lab where the recording was carried out (conditions described below). Thereafter, the insects were starved for one hour inside 1.5ml aerated tubes, then anesthetized with CO_2_ for 5 seconds. Individuals were immobilized with a vacuum device (Eyela Aspirator A3S; Rikakikai, Tokyo, Japan) and tethered. The tip of an 18 μm-gold wire, 3 cm long was placed on the insect pronotum, and glued with a double layer of silver conductive paint (Ted Pella, no. 16034; Pelco^®^ Colloidal Silver, Ted Pella, Redding, CA, USA). The tip of the wire was bent in order to create a loop that enhanced the resistance of the connection. The other end of the wire was attached to a copper electrode measuring 3 cm in length × 1 mm in diameter, using silver paint. After the wiring, the silver paint was allowed to dry and the insect was allowed to recover for approximately 10 minutes, securing the tip of the electrode to a polystyrene support. The insect was left crawling over a piece of filter paper to check if the wiring was strong enough to resist jumping and crawling. Thereafter, the electrode was plugged into the EPG head stage, with the insect left dangling over the plant without touching it for ca. five minutes before placing it on the plant. The soil copper electrode (10 cm long × 2 mm wide) of the EPG device was then inserted into the pot substrate. The system was assembled inside a Faraday cage, in an acclimatized room (23 ±2°C).

Feeding behavior was recorded for 8 hours with a Giga 8-DC EPG (EPG-systems, Wageningen, The Netherlands) at 1 Giga Ohm input resistance. Output from the EPG at 100x gain was digitalized at a rate of 100 samples per sec. per channel, and recorded using Stylet+ software (EPG-systems, Wageningen, The Netherlands). Substrate voltage was adjusted following the calibration instructions of the DC EPG equipment so that EPG output signals fit into the +5V to -5V window provided by the software. In order to determine the electrical origin of the waveform, voltage adjustment to positive and negative levels were performed in different periods for each waveform to find out if they were either of R or emf origin. Waveforms that are pure R become inverted when changing from a positive to a negative output voltage. In contrast, pure emf signals are insensitive to output voltage adjustments [32]. Waveforms amplitude and frequency were estimated based on the average of 60 observations for each type of waveform (three observations per recorded insect).

### Data analysis and further experimental design

After identifying the typical waveform categories of *P*. *spumarius* on olive, we calculated a series of sequential and non-sequential variables of the EPG recordings. The non-sequential variables were: 1) (average) number of waveform events per insect (NWEI); 2) (average) waveform duration per event per insect (WDEI); 3) (average) duration of each waveform per insect (WDI). The sequential variables were: 1) time to the first xylem contact after the beginning of the recording; 2) time to the first xylem ingestion after the beginning of the recording; 3) time to the first xylem contact after the beginning of the probe; 4) time to the first xylem ingestion after the beginning of the probe; 5) time to the first sustained xylem ingestion (that we conventionally considered as a xylem ingestion event longer than 10minutes) after the beginning of the recording; 6) time to the first sustained xylem ingestion after the beginning of the probe. EPG data were elaborated with a novel Excel Workbook developed purposely for *P*. *spumarius* by Antonio J. Alvarez (Universidad de Almeria, Spain). The typical sequence of waveform events during stylet penetration was assessed by calculating the likelihood of a certain waveform being followed by another waveform type (transitional probabilities). Only sequences of events with probabilities higher than 2% were considered [33, 34].

Furthermore, additional EPG recordings were carried out to assess sex-related differences of *P*. *spumarius* feeding behavior on olive plants. Thus, the feeding behavior of 15 males on olive plants was recorded by EPG in parallel with females (females used for feeding behavior characterization experiment), using the same procedure and conditions described above for *P*. *spumarius* females. Differences in the EPG variables recorded were analyzed by non-parametric Wilcoxon rank sum test with continuity correction. Statistical analysis was performed with the software R 3.3.3 [35].

### Simultaneous EPG and video recording of *P*. *spumarius* excretion

EPG waveforms were correlated with adult *P*. *spumarius* excretory behavior by running simultaneous EPG and video recording following a methodology similar to that described by Powell et al. [36]. Eight spittlebug adults (4 females and 4 males) feeding on olive were used; we carried out observations on excretion behavior with non-starved individuals since starvation may cause anomalous excretory behavior [37]. Tethering of *P*. *spumarius* and connection to the EPG device was performed as described above. For video recording, we used a Dino light eyepiece (Dino-Lite Europe, The Netherlands) installed on a Kyowa stereomicroscope (Kyowa Optical, SDZ-PL). The video was acquired simultaneously with the EPG signals through Dino Capture 2.0 software (Dino-Lite Europe, The Netherlands), manually synchronizing the two devices, and stored for later analysis and comparison with the EPG waveforms. Furthermore, *P*. *spumarius* excreta were collected either on the video-recorded individuals, or on ten additional spittlebugs (five males and five females) feeding on olive plants, through a piece of parafilm stretched beneath the stem where the insect was feeding. Thereafter, presence/absence of aminoacids in *P*. *spumarius* excreta was assessed by reaction of collected fluid with ninhydrin 2mg/ml of ETOH 96% [38]. Quantitative sex-related differences in excretion between males and females and correlation between waveforms and excretion frequencies were calculated by Wilcoxon rank sum test with continuity correction and Pearson’s product moment correlation, respectively. Statistical analysis were performed with the software R 3.3.3 [35].

### Simultaneous EPG and video recording of *P*. *spumarius* nymphs excretion/secretion

*P*. *spumarius* nymphs spend their life-time within a characteristic foamy case (termed “spittle”), composed of secretions produced by abdominal glands mixed with fluid voided from the anus [39]; therefore, the production of the spittle indicates ongoing ingestion. In order to collect further evidences about the biological meaning of the EPG waveforms presumably related to xylem ingestion, the feeding behavior of nymphs connected to the EPG device was simultaneously video recorded, using the same methodology and equipment described above for adult excretion. *S*. *olearaceus* was used as host plant for the EPG. Furthermore, we attempted to observe and describe the movements of cibarial muscles during feeding visible through the tender and translucent nymphal cuticle, correlating them with the corresponding waveform, following an approach similar to Dugravot et al. [40]. Nymphs were collected and reared as described above. The cages containing the *S*. *oleraceus* plants with the nymphs were moved for acclimation to the room where the EPG experiments were carried out two days before the recording. For the EPG, nymphs of the third and fourth instars were used. The spittle residual embedding the body was removed with a paintbrush wet in a solution of ETOH 25% and Triton X100 1% [41]; then the individuals were anesthetized by exposure to 4°C for 20 minutes. A golden wire of 18 μm was connected to the abdomen perpendicularly to the body, with a drop of silver conductive glue under a magnifying lens. Nymphal feeding behavior was recorded with a Giga-4 EPG device gain 75x (EPG-systems, Wageningen, The Netherlands). Abdominal and anal tube movements and anal excretions of nymphs, as well as cibarial muscle movements when visible, where correlated with the EPG waveforms observed.

### Micro-CT histological studies and correlation of stylets tips position with EPG waveforms

**Fig 1:**
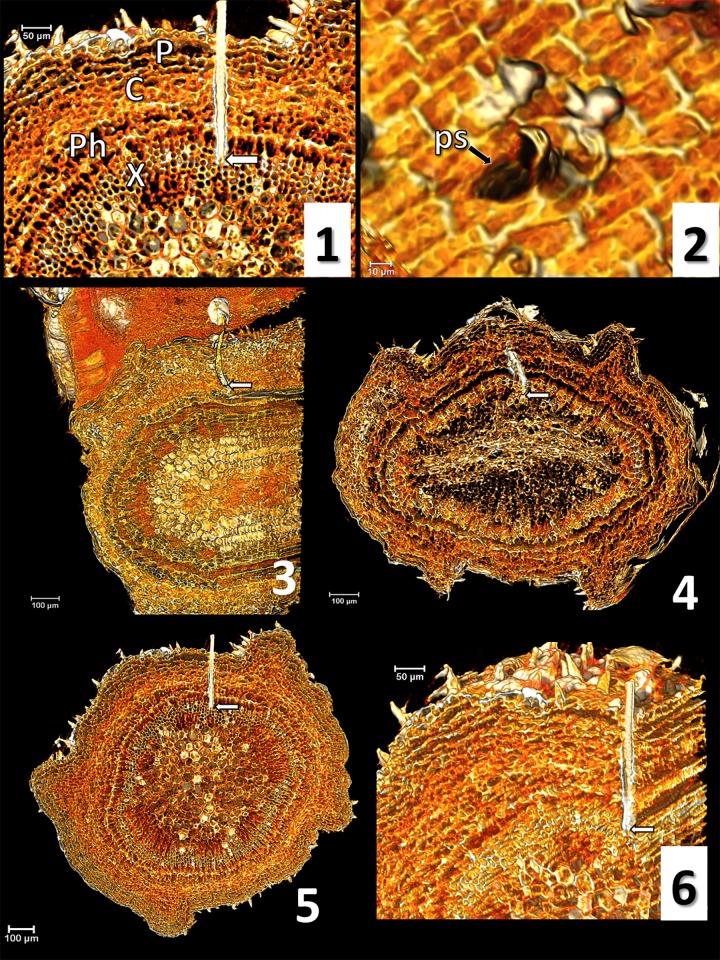
Micro-CT volume rendering reconstruction images and correlation waveform/stylets position. 1) olive anatomy: P (periderm), C (cortex), Ph (phloem), X (xylem) (nomenclature according to Ruiz et al. [45]); 2) probing site (ps); 3) waveform C; 4) waveform Xc; 5) waveform Xi; 6) Xi, lateral view. White arrows indicate the stylets tip.

**Fig 2:**
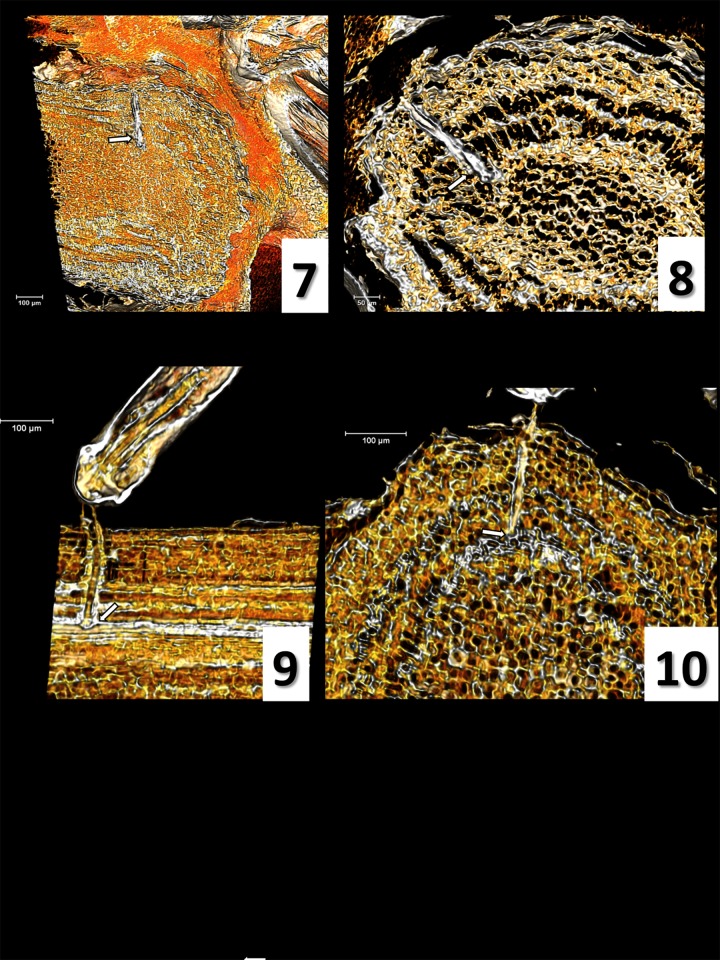
Micro-CT volume rendering reconstruction images and correlation waveform/stylets position. 7) waveform R general view; 8) R, detailed view; 9) waveform N, lateral view including the *labium*; 10) waveform N. White arrows indicate the stylets tip.

A green succulent olive young stem (three months old) was secured to a plastic Petri dish using modelling clay, leaving a 5 cm portion exposed to *P*. *spumarius* feeding. When a given waveform of interest was observed, liquid nitrogen was gently poured over the stem close to the feeding site to fix the stylets within the plant tissue as fast as possible. The dish containing both the insect and the plant was transferred into a polystyrene box containing dry ice for 10 min. Then *P*. *spumarius* legs were secured to the stem using transparent nail polish, prior to dehydrating the sample using ETOH 96% at -20°C for 48 hours. Thereafter, the sample was stained with 1% iodine in ETOH 96% for 48 hours at 4°C, and successively dehydrated into hexamethyldisilazane (HDMS) for 24 hours at room temperature. Stylet position during each specific waveform of interest was assessed by computer microtomography (micro-CT) [42]. Samples were scanned with a SkyScan 1172 high resolution microtomographer, upgraded to have a Hamamatsu 100/250 source and a SHT 11Mp camera. The scanning parameters were setup as it follows: Isotropic voxel size = 1 μm (Fig 1: a, b, e, f), 1.76 μm (Fig 1: c, d), and 1.9 μm (Fig 2: c, d); Source Voltage = 59KV, Source Current = 49μA, Rotation step = 0.3°, 360° of rotation scan, and no filter. The reconstruction of raw image dataset and cleaning were performed using the Bruker micro-CT Skyscan software (NRecon, DataViewer, and CTAnalyser, www.skyscan.be), following the methodology described in detail previously [43]. The Amira software v.6.4 was used for volume rendering images (Amira 3D Visualization and Analysis Software.v.6.4. Hillsboro, Oregon, USA: FEI; 2017). Ultimately, we considered only the images produced from those spittlebug samples with no visible insect displacement.

## Results

### EPG waveforms of *P*. *spumarius* adults

**Fig 3:**
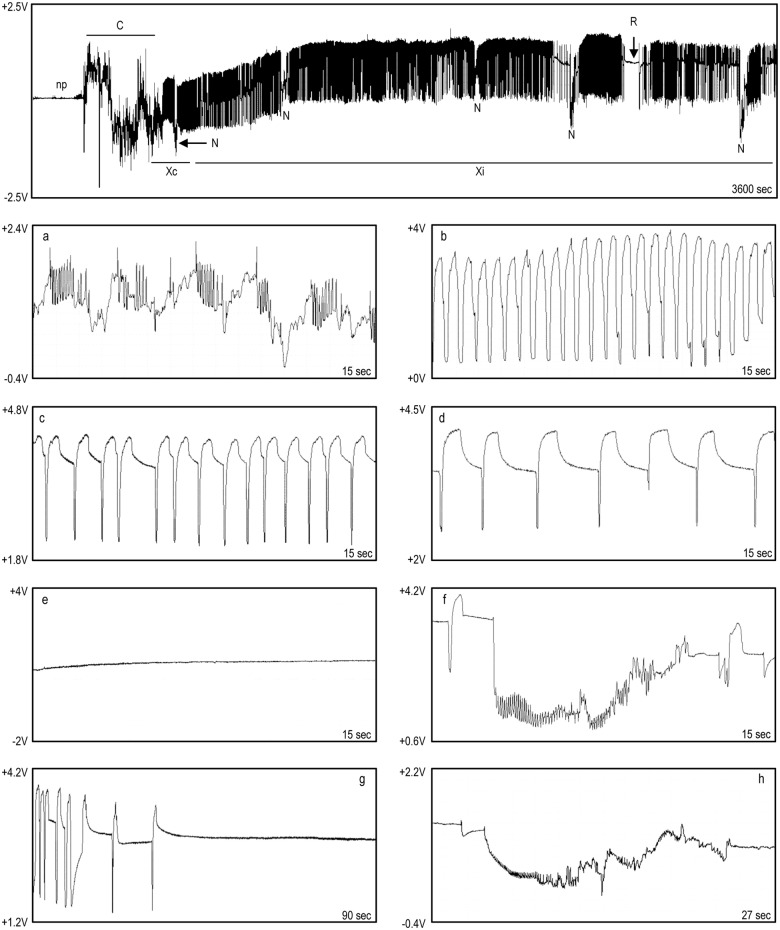
EPG recording for *P*. *spumarius* on olive. Non probing (np), waveform C followed by Xc and Xi, with N interspersed, and flat waveform R. Below the general view, close-ups of the waveforms: a) C; b) Xc; c) Xi, high frequency; d) Xi, low frequency; e) R; f) N; g) transition Xi/R; h) drop similar to N during R.

**Fig 4:**
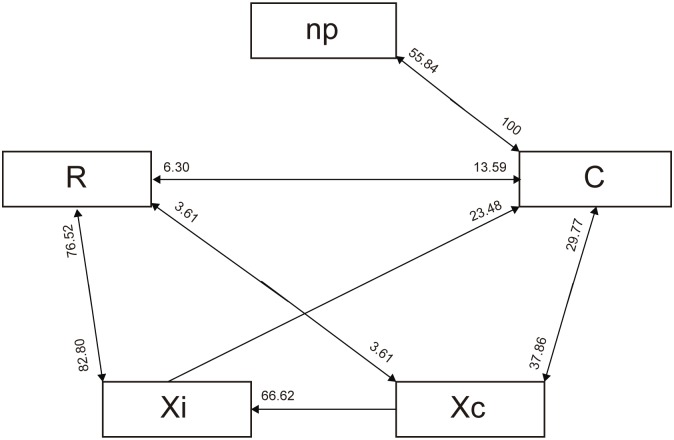
Transition scheme of waveforms events for *P*. *spumarius* on olive cv Picual during the 8 hours EPG (likelihood of waveforms events). The values near the arrows correspond to the likelihood of a certain waveform being followed by another waveform type. Probabilities <2% are not shown. Waveform N was not represented in the diagram, because it occurs as brief interruption within waveforms Xc and Xi (and likely R).

**Fig 5:**
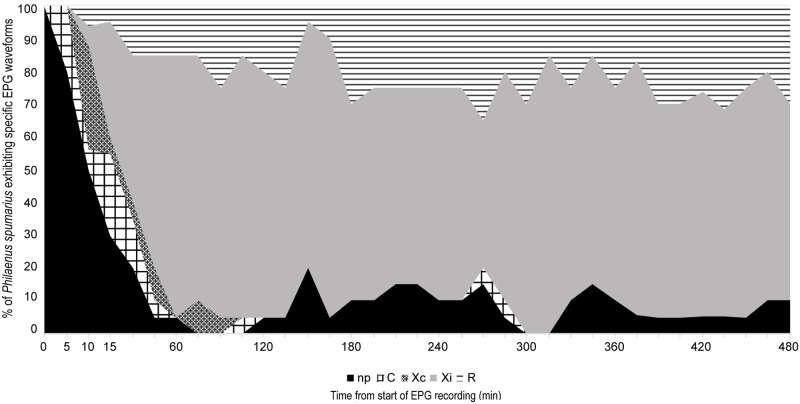
Temporal development of stylets activity for female *P*. *spumarius* on olive.

For *P*. *spumarius*, five main waveforms were identified and named following almost the same terminology as Miranda et al. [44], according to their high similarity with the ones reported for *Bucephalogonia xanthophis* Berg (1879) (Hemiptera: Cicadellidae): C (corresponding to S in *B*. *xanthophis*), Xc, Xi, R and N (Fig 3). Waveform C stands for pathway, including both stylet penetration and withdrawal; Xc and Xi represent xylem contact/pre-ingestion and active xylem ingestion, respectively; R indicates resting occurring during xylem activity; N represents an interruption during xylem phase. The five waveforms were observed in all of the recordings. No changes in waveforms characteristics were observed when the polarity of the substrate voltage was switched during the recording, indicating that the main electrical components of these waveforms have an electromotive force (emf) origin. The main waveforms characteristics of *P*. *spumarius*, and their biological meaning are reported in Table 1. Considering the most likely succession of events (Fig 4), and the temporal development of stylet activity (Fig 5), *P*. *spumarius* probe begins with waveform C, followed by Xc and Xi of either low or high frequency. Waveform Xi alternates with R during the probe. N may occur either during Xc and Xi throughout the whole probe; furthermore, interruptions similar to N occur also during R (these last ones were not included in the analysis). The values for the main sequential and non-sequential EPG variables of *P*. *spumarius* males and females are reported in Table 2.

**Table 1:** Waveforms characteristics and biological meaning.

Waveforms characteristics						Correlation		
Waveform	Amplitude % (V)		Frequency (Hz)	Voltage level	Electrical origin	Stylet tips in plant tissue	Excretion	Activity
**C**	8.94% (0.447)		mixed	e/i	emf/R	All tissues	no	Secretion of salivary sheath/ intracellular penetration
**Xc**	42.76% (2.138)		Waves: 1.6 (0.7–2.9)	e	emf	Xylem	no	Unknown/ Supposed xylem contact
			Peaks: 1.7 (0.8–2.9)					
**Xi**	31.06% (1.553)		Waves: 0.7 (0.3–1.5)	e	emf	Xylem	yes	Ingestion
			Peaks: 0.6 (0.1–1.5)					
**N**	**1st drop**	**N**	mixed	e/i	emf	Xylem	no	Unknown/ Interruption during xylem phase
	48.28% (2.414)	6.36% (0.318)						
**R**	12.7% (0.635)		<0.1	e	emf	Xylem	no	Unknown/ supposed resting

5V = 100% amplitude

***Abbreviations:*** e = extracellular; i = intracellular; emf = electromotive force; R = resistance

**Table 2:** Non-sequential and sequential EPG variables of adults of *P*. *spumarius* (males and females) on olive. Time is expressed in minutes.

**Females on olive**								
**Waveform**	**Non sequentials**						**Sequentials**	
	**n**	**NWEI**		**WDEI**	**WDI**		**Time for a certain event to occur**	**mean±se (min-max)**
		**mean±se**	**min-max**	**mean±se**	**mean±se**	**min-max**		
**np**	20	4.4±0.64	1–11	19.1±9.66	51.14±12.8	2.9–199.5	Time to first Xc from the beginning of the EPG	17.23±4.12 (2.3–74)
**C**	20	6.15±0.99	1–16	2.47±0.37	10.52±1.9	0.7–28.7	Time to first Xi from the beginning of the EPG	21.91±4.24 (4.2–75.5)
**Xc**	20	2.85±0.49	1–7	1.49±0.32	4.74±0.83	0.9–16.1	Time to first Xc from the beginning of the probe	8.74±3.35 (0.9–63.8)
**Xi**	20	8.55±1.27	3–29	150.66±36.02	311.81±27.37	86–463	Time to first Xi from the beginning of the probe	13.41±3.59 (2.4–65.2)
**N**	20	20.3±2.07	2–35	0.2±0.02	4.25± 0.68	0.15–8.73	Time to the first Xi>10min from the beginning of the EPG	39.52±7.049 (4.3–129)
**R**	20	7.25±1.35	1–29	26.62±5.27	101.76±21.91	3–318.3	Time to the first Xi>10min from the beginning of the probe	31.02±7.23 (3.2–127.8)
**Males on olive**								
**Waveform**	**Non sequentials**						**Sequentials**	
	**n**	**NWEI**		**WDEI**	**WDI**		**Time for a certain event to occur**	**mean±se (min-max)**
		**mean±se**	**min-max**	**mean±se**	**mean±se**	**min-max**		
**np**	15	4.46±0.82	1–13	26.99±6.09	108.20±29.99	1.4–356.3	Time to first Xc from the beginning of the EPG	31.3±6.09 (2.50–71.3)
**C**	15	6.26±1.56	1–24	3.53±0.70	17.39±5.96	1–81.7	Time to first Xi from the beginning of the EPG	43.29±8.08 (5.70–90.50)
**Xc**	15	2.6±0.61	1–10	1.81±0.59	4.94±1.09	0.9–17.8	Time to first Xc from the beginning of the probe	10.98±4.26 (1–65.60)
**Xi**	15	10.8±1.67	2–22	113.2±36.42	199±29.88	15.6–404.2	Time to first Xi from the beginning of the probe	23±7.06 (2.5–83.8)
**N**	15	14.4±2.37	3–34	0.25±0.02	3.6±0.77	0.9–9.43	Time to the first Xi>10min from the beginning of the EPG	67.57±7.98 (9.1–107)
**R**	15	10.47±1.74	1–22	60.69±15.9	150.5±27.33	20.8–401.4	Time to the first Xi>10min from the beginning of the probe	47.27±7.56 (9–105.8)

NWEI: number of waveform events per insect; WDI: waveform duration per insect; n: number of insects performing the waveform out of the total tested (20 females on olive, 15 males on olive, 13 individuals for vetch).

### Waveform C

Waveform C (Fig 3: a) was always the first during the probe (stylet penetration in the plant). This waveform was characterized by an irregular trace, with great oscillations in frequency and amplitude. Furthermore, waveform C occurred at variable voltage level (either positive or negative) lower than those observed for the other waveforms, indicating intra/intercellular penetration.

### Waveform Xc

The sinusoidal waveform Xc (Fig 3: b) occurred between C and Xi, and was characterized by a regular trace of relatively high amplitude peaks (mean value of ca. 2V), with a frequency ranging 0.8–2.9 Hz. This waveform showed an extracellular (positive) voltage level, and a mean duration of <5min.

### Waveform Xi

Waveform Xi (Fig 3: c and d) had a regular trace characterized by peaks and plateaus, with a mean amplitude of ca. 1.5V. Oscillations in peaks frequency occurred during Xi, with an alternation of lower (0.1–0.5Hz) and higher (0.8–1.5Hz) values (in Fig 3 both higher frequency and lower frequency Xi are shown, images c and d, respectively). Voltage level of waveform Xi was positive in all the observed cases, indicating extracellular activity. Considering the overall time spent in probing activity during the 8 hour recordings on olive, 72.7% for females and the 52.53% for males was spent on waveform Xi.

### Waveform R

Waveform R (Fig 3: e and g) generally occurred after Xi, alternating with the latter during the probe. It was characterized by a flat trace, with very low amplitude and frequency, and random oscillations with a frequency lower than 0.1Hz.

### Waveform N

Waveform N (Fig 3: f) represents a potential drop occurring during Xc or Xi; moreover, drops similar to N were observed also during waveform R (these last one were not included in the analysis) (Fig 3: h). The voltage level indicates an extracellular activity, although sometimes it dropped to negative values. Both frequency and voltage, the latter displaying a mean value for the drop of 2.4V, and of ca. 0.3V for the rest of the waveform, were highly variable.

### Sex-related differences

Regarding sex-related differences of *P*. *spumarius* feeding on olive, statistical analysis of the sequential variables revealed that males take significantly longer time to begin xylem ingestion phase once alighted on the plant (time to first Xi from the beginning of the EPG: W = 207.5, p = 0.025; time to the first Xi>10 min from the beginning of the EPG: W = 218, p = 0.008). However, once the probe has started required times to perform the above-mentioned activities did not differ between the two sexes. Considering non-sequential variables, by analyzing WDI we found that females spent significantly more time in sustained xylem ingestion (Xi) than males, with a mean value for males and females of 199 and 311 minutes, respectively (W = 77.5; p = 0.016). Regarding WDEI, significant differences were found in non-probing and resting R, with single events significantly shorter in females compared to males (W = 82, p = 0.02 for np; W = 88.5, p = 0.042 for R). Finally, considering NWEI, no differences were found between males and females. Furthermore, *P*. *spumarius* on olive made an average of 4–5 probes during the 8h of recording (4.13±0.76 for males, 4.85±0.68 for females), with no significant sex-related difference.

### The timing and nature of *P*. *spumarius* excretions when feeding on olive

Excretion was observed on four out of the eight EPG/video-monitored individuals, two males and two females. Prior to start of excretion, *P*. *spumarius* tended to cross the anal tube over the last abdominal segments; excretion occurred with the anal tube bent downward, and the drop was rapidly released. We observed excretion two to ten minutes after the beginning of Xi; nevertheless, during low frequency Xi (0.1–0.2 Hz) excretion was not observed. Females excreted significantly more than males, with mean values of 6.94±0.97 drops per minute for females, and 3.57±0.59 drops per minute for males (W = 55.5, p-value = 0.011). In males, the frequency of ingestion waveform was not correlated with the excretion frequency, according to Pearson’s product moment correlation (t = -0.062; p = 0.951); on the contrary, the ingestion waveform and excretion frequencies were significantly correlated in females (t = 5.806; p<0.001). Overall, merging male and female data, ingestion and excretion frequencies were significantly correlated (t = 2.212; p = 0.035), although the two events were not synchronous (an ingestion waveform (Xi) peak does not correspond to a drop). We did not observe excretion during pathway C, Xc, R or N. *P*. *spumarius* occasionally performed during the probe exaggerated abdominal movements, during which excretion did not occur. Additionally, ninhydrin never reacted with the excreta collected from the parafilm at the end of the recording, neither for the video recorded individuals nor for the ten insects not video recorded, suggesting that *P*. *spumarius* ingested only or almost exclusively xylem sap.

### Observations on *P*. *spumarius* nymphal excretion/secretion

Twelve EPG recordings, from 30 minutes to 1 hour long were obtained. It was not possible to make longer recordings because the production of spittle gradually led to a loss of electrical connection between the wire and the insect, with the appearance of a flat (non-probing) waveform despite the stylets being visibly inserted in plant tissues. The waveforms of nymphs were similar to those of adults and the same C, Xc, Xi, and N waveforms were observed (data not shown). We observed excretion from the anus and secretion from abdominal glands occurring during waveform Xi. Fluids voided from the anus and secreted by the glands were mixed and spread over the entire body by mid legs and body movements. When excretion or secretion occurred, no exaggerated abdominal movements were evident. Abdominal movements (lateral or telescopic) and ingestion waveform frequencies were significantly correlated according to Pearson’s product moment correlation (t = 4.111, p< 0.001), although the two events were not perfectly overlapping (each peak of the ingestion waveform does not correspond to an abdominal movement). Moreover, abdominal and anal tube movements without excretion or secretion were observed even during the pathway phase (waveform C). Additionally, in one out of twelve observed cases, we directly observed (by means of the stereomicroscope and video recording) the cibarial muscle movements through the insect cuticle while the nymph was feeding. Interestingly, we noticed synchrony between cibarial muscle movements and ingestion waveform (Xi) peaks and plateaus. A complete muscle movement corresponded to one peak and one plateau. The muscle relaxation and diaphragm collapse that push the fluid toward the esophagus corresponded to one plateau, while the muscle contraction and cibarial diaphragm lift corresponded to one peak of the xylem ingestion waveform Xi (S1 Video shows in parallel nymph’s cibarial muscle movements and the simultaneous EPG signals recorded).

### Micro-CT of probed tissues and correlation with EPG waveforms

Images produced through micro-CT are shown in Figs 1 and 2 (nomenclature according to Ruiz et al. [45]). Initially, *P*. *spumarius* secretes a small amount of sheath saliva on the stem surface, creating a salivary flange that marks stylet insertion in plant tissue (or probing site, Fig 1: 2). After crossing the epidermis (periderm), the stylets move intracellularly straight across the parenchyma (cortex), the phloem, and eventually reach xylem vessels (Fig 1: 3 and 4). Indeed, the sample collected during waveform C clearly shows the stylet crossing straightforward through the parenchyma tissues moving toward the vascular bundle (Fig 1: 3). In both Xc and Xi (one sample selected per each of the two waveforms), the stylet tips terminate in a xylem vessel (Fig 1: 4, 5, 6). In the two samples collected while the insect was performing the flat waveform R, the images show stylet tips ending in xylem (Fig 2: 7 and 8). Also during waveform N, in all of the three samples selected and analyzed, we observed the stylets inside a xylem vessel (Fig 2: 9 and 10). Finally, S2 Video represents a series of micro-CT images showing the precise position of stylets during plant penetration of *P*. *spumarius* on an olive petiole at the time that waveform Xi was recorded.

## Discussion

The waveforms observed and correlated with feeding phases for *P*. *spumarius* were very similar to those described for *B*. *xanthophis* by Miranda et al. [44] using the same type of EPG device (DC-Giga amplifier).

Waveform C represents stylet penetration activities during the pathway phase, salivation and build-up of the salivary sheath, and tissue exploration while moving toward the xylem vessels. In contrast with Miranda et al. [44], we did not consider penetration and withdrawal as different waveforms, as we considered both activities as part of waveform C.

Waveform Xc indicates the first contact with a xylem vessel and could represent a pre-ingestion or trial ingestion (term previously proposed by Crane [46] for *Graphocephala atropunctata* Signoret (Hemiptera: Cicadellidae) (previously *Hordnia circellata*)). The images produced by micro-CT indicate that during this waveform the stylets are within a xylem vessel. Xc waveform is characterized by a high frequency sinusoidal shape, in contrast with the alternation of peaks and plateaus that occurs during waveform Xi. During Xc, no excretion was observed and we have no conclusive evidence about the biological meaning of this waveform. Besides the high similarity of Xc with that described by Miranda et al. [44], this pattern also resembles the C1 waveform, representing the last waveform in pathway phase described for *G*. *atropunctata* and *Homalodisca vitripennis* Germar (1821) (previously *H*. *coagulata*) (Hemiptera: Cicadellidae) using an AC-EPG system [34, 47]. In the latter work, C1 was sometimes correlated with xylem contact. We suggest, consistently with Miranda et al. [44] that Xc represents an activity in response to mechanical or chemical stimuli resulting from the contact with a xylem vessel. During Xc, once reached the xylem, the insect could taste the sap by means of the pre-cibarial sensilla [48] without swallowing it, and initiates cibarial pump activity to balance the vessel tension in order to later begin the proper feeding (active xylem sap ingestion). However, this hypothesis should be further tested in order to definitely determine the biological meaning of waveform Xc.

Waveform Xi corresponds to active xylem sap ingestion, as proven by occurrence of excretion in adults and excretion/secretion in nymphs only during this EPG pattern. The production of the first excretory droplets was delayed in respect to the beginning of the Xi waveform (two to 10 min). Likely, the delay could be associated with the fact that excretion by *P*. *spumarius* requires previous accumulation of xylem sap in the gut to process, as further proven by the absence of excretion at Xi frequency as low as 0.1–0.2 Hz, thus in correspondence to a likely slow and reduced sap intake. The amount of excreta produced by *P*. *spumarius* during our observations (6.94 drops/min for females, 3.57 for males) is consistent with the data reported by Malone et al. [18] (ca. 1 drop every 10–11 sec), and far lower than values described for sharpshooters such as *B*. *xanthophis* (ca. 24 drops/min, [44]), and *G*. *atropunctata* (ca. 1drop/sec [34]). Therefore, although only indirectly, we hypothesize that *P*. *spumarius* has a lower ingestion rate than sharpshooters.

Furthermore, through the delicate nymphal cuticle, we also observed the movements of cibarial muscles and diaphragm, correlating each rhythmic movement to the Xi waveform pattern (S1 Video). According to our video recordings, we can propose that each peak that composes Xi corresponds to muscle contraction and diaphragm lift that create the required tension for sap aspiration, while each plateau corresponds to muscle relaxation and diaphragm collapse, which lead to sap swallowing. A similar correlation was described for *H*. *vitripennis* with the flow of Chinese ink particles suspended in artificial diet observed moving inward/outward the stylet [49].

*P*. *spumarius* is rapid in reaching the xylem and initiating sap ingestion (Fig 5), with first Xc and Xi occurring 8.74 and 13.41 minutes after the onset of the first probe, respectively (mean values calculated for females, and not statistically different from those of males) (Table 2). As remarked in several studies [15, 16, 17, 18, 19, 20], and confirmed here by analysis of the excretion process and the non-reaction of excreta with ninhydrin, *P*. *spumarius* ingests only xylem sap. Nevertheless, since the meadow spittlebug has been reported as a vector of the Elm yellow phytoplasma, and the latter organism only colonizes the phloem [50, 51, 52], in certain conditions and on certain hosts, the insect might divert its behavior from xylem to phloem feeding. For example, significant changes in feeding behavior have been related to plant susceptibility/resistance in *Nephotettix virescens* Distant (1908) (Hemiptera: Cicadellidae), a phloem feeder that tends to feed more often from xylem in resistant rice varieties [53]. Our EPG recordings were restricted to the first 8 hours, thus any further feeding activities happening after that time were not recorded. Nevertheless, prior to start the feeding behavior characterization study described in this paper, we performed two comparative 24-hours EPG recordings, one with two males, the other with two females (data not shown). Briefly, two individuals of the same sex were tethered and connected to the EPG as described above. The electric circuit was closed only for one of the insect, while the other was not subjected to electric current, since the plant electrode was not connected to the potting media. After the 24 hours EPG, each individual was caged singly on a vetch plant, transferred to a growth chamber at 25°C, 80% HR, and monitored at intervals of eight hours for three days. Either the two males or the two females, whether subjected or not to EPG electric current, were found to be alive and active at the end of the three days monitoring periods. Furthermore, neither phloem-related waveforms nor waveforms other than those described for the eight hour recordings were observed.

Throughout the recording, periods of sustained xylem ingestion (Xi) were interspersed with periods of resting, defined by waveform R, also present in the last 2 hours of recording (Fig 5). During this waveform we observed no activities, neither fluid excretion nor movements, while the stylets remained within the xylem (Fig 2: 7 and 8); the same resting behavior for the meadow spittlebug has been described by Malone et al. [18]. The slight gradual voltage increase occurring during waveform R could be associated with a tiny partial withdrawal of stylets, as observed also by Joost et al. [49]. Voltage variation during R, with occasional drops and spikelet bursts, may be associated with stylet movements within the same sheath, before restarting sustained ingestion, as well as other activities or tentative ingestion. Therefore, while Miranda et al. [44] suggested that this R waveform was a resting behavior occurring in the parenchyma before reaching any vascular tissues, we propose that R represents a resting phase during the xylem ingestion period, required by *P*. *spumarius* after spending a great amount of energy in loading the cibarial pump during the xylem ingestion phase [54, 55].

Finally, waveform N, consistently with Miranda et al. [44], represents an interruption during xylem phase, occurring either during Xc or Xi; drops similar to N, that we did not consider in the analysis and deserve further dedicated investigation, were also sometimes observed during resting R (Fig 3: h). Through micro-tomography, we assessed that during this specific waveform the stylet tips are located within the xylem. Therefore, if N is an activity performed in a xylem vessel, the initial drop (Fig 3: f) should not be associated with a potential drop related to an stylet puncture of a living cell (transmembrane potential) as reported for stylet cell punctures made by aphids, but could be more likely generated by streaming potentials derived from valve movements [26, 32]. These valve movements could represent extravasation or egestion of fluids from the cibarium/pre-cibarium to the food canal and then to the plant tissues, but further investigation is needed to test this hypothesis [56, 57]. At the same time, N could even represent an interruption in xylem phase and the successive switch to another xylem vessel, as suggested by Backus et al. [47] and Miranda et al. [44]. Further research is also needed in order to characterize the biological meaning of the different sub-phases of the N waveform (Fig 3: f and h), looking for a possible correlation with waveforms XC and XN in sharpshooters, thoroughly described by Backus [27].

Considering the overall 8-hours of EPG recording, the main sex-related differences for *P*. *spumarius* feeding on olive were the time required to begin xylem ingestion, and the duration of xylem ingestion. Males require longer time for the onset of xylem ingestion after alighting on the plant, while this difference was not statistically significant once the time to the first Xi was calculated from the beginning of the probe. One of the explanations underlying the observed difference could be the greater sensitivity of males to manipulation, with longer recovering times after tethering. Regarding the duration of Xi, *P*. *spumarius* females ingest longer than males, as also confirmed by the greater amount of excreta produced by the former. Overall, females spend ingesting (Xi waveform) the 72.7% of the probing time, consistently with data produced on grape by Sandanayaka et al. [30]. On the contrary, we observed no sex-related differences in the number of probes performed. As demonstrated by Krugner et al. [58] for *H*. *vitripennis*, transmission efficiency of *X*. *fastidiosa* is independent from the sex of the vector. Therefore, the fact that the main difference in feeding behavior between male and female is the duration of the ingestion phase, suggests that bacterial transmission could be driven by a mechanism dissociated from the time spent in xylem ingestion. Conversely, the number of probes performed by a vector was found to be positively correlated with transmission efficiency [59, 60]. According to Almeida and Backus [34], *G*. *atropunctata*, accomplished from five to 27 probes in 20 hours EPG recordings on grapevine. *P*. *spumarius* instead made an average of 4–5 probes during the 8h recording on olive. The development of *X*. *fastidiosa* systemic infections is directly related to the number of inoculation events, thus to the number of probes performed by the vector [60]. Therefore, the reduced number of probes in *P*. *spumarius* compared to *G*. *atropunctata* could explain the reported lower *X*. *fastidiosa* transmission efficiency to grapevine by the former in respect to the latter [31, 61]. However, it has to be taken into account that the two feeding behavioral studies were performed on two different host plants (grapevine for *G*. *atropunctata*, olive for *P*. *spumarius*), thus any hypothesis should be further tested under the same experimental conditions.

Despite the fact that several research efforts have led to significant breakthroughs in the direction of understanding vector-bacterium relationship [27, 62, 63, 64], conclusive evidences about the transmission mechanism of *X*. *fastidiosa* by its major vectors are still missing [6]. Furthermore, currently available data are derived exclusively from studies on sharpshooter-bacterium interaction. Although *X*. *fastidiosa* transmission dynamics by *P*. *spumarius* seems to be similar to that reported for sharpshooters, the bacterium-spittlebug interaction presents elements of novelty. Indeed, the meadow spittlebug hosts a bacterial population within the foregut relatively low when compared to sharpshooters [14; 61]. This characterization of *P*. *spumarius* feeding behavior represents a first step in order to understand the relationship between the spittlebug and *X*. *fastidiosa*. More research efforts are urgently needed in order to detect the precise behaviors associated with acquisition and inoculation of the fastidious bacteria by the meadow spittlebug. Such data might open new venues for developing environmentally sustainable control of *X*. *fastidiosa*-mediated diseases, through the disruption of the transmission mechanism.

## Supporting information

S1 Video Correlation between cibarial pump movement and related EPG waveform (Xi). (MP4)

S2 Video Video obtained from micro-CT showing *P*. *spumarius* stylets in xylem vessel. (MKV)
